# Protocol for the management of psychiatric patients with psychomotor agitation

**DOI:** 10.1186/s12888-017-1490-0

**Published:** 2017-09-08

**Authors:** Eduard Vieta, Marina Garriga, Laura Cardete, Miquel Bernardo, María Lombraña, Jordi Blanch, Rosa Catalán, Mireia Vázquez, Victòria Soler, Noélia Ortuño, Anabel Martínez-Arán

**Affiliations:** 1 Hospital Clínic de Barcelona, Institute of Neuroscience, Barcelona, Catalonia Spain; 2 University of Barcelona, Barcelona, Catalonia Spain; 3 Biomedical Research Institute August Pi i Sunyer (IDIBAPS), Barcelona, Catalonia Spain; 4 Center of Biomedical Network Research on Mental Health (CIBERSAM), Barcelona, Catalonia Spain; 5 Numancia Salut Mental, Parc Sanitari Sant Joan de Déu, Barcelona, Catalonia Spain

**Keywords:** Inhaled loxapine, Psychomotor agitation, Protocol, Verbal de-escalation, Physical restraint

## Abstract

**Background:**

Psychomotor agitation (PMA) is a state of motor restlessness and mental tension that requires prompt recognition, appropriate assessment and management to minimize anxiety for the patient and reduce the risk for escalation to aggression and violence. Standardized and applicable protocols and algorithms can assist healthcare providers to identify patients at risk of PMA, achieve timely diagnosis and implement minimally invasive management strategies to ensure patient and staff safety and resolution of the episode.

**Methods:**

Spanish experts in PMA from different disciplines (psychiatrists, psychologists and nurses) convened in Barcelona for a meeting in April 2016. Based on recently issued international consensus guidelines on the standard of care for psychiatric patients with PMA, the meeting provided the opportunity to address the complexities in the assessment and management of PMA from different perspectives. The attendees worked towards producing a consensus for a unified approach to PMA according to the local standards of care and current local legislations. The draft protocol developed was reviewed and ratified by all members of the panel prior to its presentation to the Catalan Society of Psychiatry and Mental Health, the Spanish Society of Biological Psychiatry (SEPB) and the Spanish Network Centre for Research in Mental Health (CIBERSAM) for input. The final protocol and algorithms were then submitted to these organizations for endorsement.

**Results:**

The protocol presented here provides guidance on the appropriate selection and use of pharmacological agents (inhaled/oral/IM), seclusion, and physical restraint for psychiatric patients suspected of or presenting with PMA. The protocol is applicable within the Spanish healthcare system. Implementation of the protocol and the constituent algorithms described here should ensure the best standard of care of patients at risk of PMA. Episodes of PMA could be identified earlier in their clinical course and patients could be managed in the least invasive and coercive manner, ensuring their own safety and that of others around them.

**Conclusion:**

Establishing specialized teams in agitation and providing them with continued training on the identification of agitation, patient management and therapeutic alternatives might reduce the burden of PMA for both the patient and the healthcare system.

## Background

Psychomotor agitation (PMA), a state of motor restlessness and mental tension, is associated with a variety of psychiatric conditions [1]. PMA may be evidenced by an increased motor activity (e.g. excessive gesturing) and emotional activation but may also be accompanied by emotional lability and a decreased level of attention and alterations in cognitive function. PMA is particularly prevalent in among the schizophrenia and bipolar disorder (BD) population [2]. In Spain, a recent report indicated that 25% of patients with schizophrenia and 15% of those with BD could be expected to suffer at least one episode of PMA each year, with a median of 2 episodes per year per patient [3].

Emergent PMA requires timely recognition, appropriate assessment and management to minimize anxiety for the patient and reduce the risk for escalation to aggression and violence that may be directed towards themselves or others [2]. Episodes of PMA may be encountered in the context of inpatient psychiatric care facilities but also in the emergency room setting and in outpatient clinics [2]. Recent studies suggest that up to 10% of all emergency psychiatric interventions are related to PMA [4–6]. Consequently, an adequate identification and management of PMA is an essential component of the care of patients with psychiatric disorders [7].

As agitation is understood as a continuum of symptoms ranging from mild to severe, [8] it is essential to detect episodes of PMA in their earliest manifestation to avoid a possible escalation of symptoms. Ineffective management of PMA might result in the unnecessary use of coercive measures (involuntary medication, physical restraint, and seclusion) that could potentially precipitate aggression or violence [9]. Moreover delayed and/or inappropriate management of PMA might lead to an increased use of hospital resources and avoidable hospitalizations with significant economic costs.

International expert consensus recommendations have been recently published for the assessment and management of patients with PMA due to their primary psychiatric condition [2]. However, there is a lack of standardized protocols and clinical tools to assist clinicians and healthcare professionals (HCP) in achieving the best possible outcome for patients presenting with an episode of PMA.

Against this background, we aimed to elaborate a protocol developed for the assessment and management of patients with PMA. The key difference with the original consensus article [2] is that it was a broad compilation of all the available bibliography on the topic that may not be always easy to apply to the daily clinical practice. Medical protocols have to be based on the current and high degree evidence literature; however, an adequate and adapted guidance to all health care systems is not always achieved from the literature perspective. Because of that, despite the current protocol is based on the same information of a recent critical review of the literature [2], it is aimed to provide specific guidance for the care of patients presented with an agitation episode adapted and specified not only to be close to clinical practice but also to be in accordance to the Spanish health care system. When used in the clinical setting, this protocol will reduce the anxiety of the patient, ensure physical safety of both patient and HCPs and minimize the risk for escalation to aggressive behaviour and violence by means of a series of standardized actions.

## Methods

This protocol represents an application and adaptation of recently published international consensus guidelines on the management of psychiatric patients with PMA [2].

The methodology consisted in a search of the most relevant articles [2], a systematic review according to the Jadad scale [10] and according to the PRISMA statement criteria [11], and a consensus among different international experts on the topic of PMA using the Delphi methodology [12]. After this international consensus was presented, main authors (EV, MG) decided to lead a new specific panel of experts in their own country to seek applicability of this protocol according to the Spanish mental health policies and laws. Using these guidelines as a foundation and the internal Standard Operating Procedures (SOP) from the Hospital Clínic i Provincial de Barcelona, a panel of Spanish mental health professionals with relevant experience in clinical guideline development and/or in systematic reviews research methodology was then convened. The expert committee consisted of eight psychiatrists, two nurses and one psychologist who were organized into subpanels based on their expertise. The purpose of the panel was to provide expert advice during the development process. A core group (EV, MG, LC) was established to design the protocol and provide more time-sensitive and operational advice according to the local policies and national laws. The core group also developed a multistep process that included an assessment of existing updated clinical guidelines, semi structured interviews, a Delphi internal consensus survey, and an external review with official national mental health scientific societies. A first round of possible evaluation and treatment recommendations, the evidence upon which they were based and the adequacy of their implementation in the Spanish healthcare system, were reviewed at a meeting of the expert panel in April 2016. Subsequently, draft recommendations were prepared by the subpanels which were then circulated to the entire group for consensus through semi structured interviews and Delphi consensus throughout 2016. Once preliminary recommendations were agreed by the expert panel, an external review was requested to the Catalan Society of Psychiatry and Mental Health as well as the Spanish Society of Biological Psychiatry (SEPB) and the Spanish Network Centre for Research in Mental Health (CIBERSAM) for input and endorsement of the recommendations included in the protocol.

The final protocol is presented in three main sections, the first section covers the initial identification and evaluation of a patient presenting with or suspected of PMA including assessment tools and differential diagnosis guidance. The second section covers recommendations for selection of appropriate interventions during an episode of PMA including environmental modifications, verbal de-escalation, pharmacological treatment and physical restraint. The third section details evaluations that should be undertaken following an episode of PMA. In addition to these main sections, three clinical algorithms have been developed to provide easy and quick guidance on the main recommendations of the protocol in relation to the initial and general management of the patient with PMA, the selection of appropriate pharmacological interventions, and standards of care when physical restraint is needed.

## Results

### Protocol 1: Identification and evaluation of psychomotor agitation

When PMA is suspected, it is recommended that the first step in the evaluation of these patients should be to ensure they are safe [2]. This may involve moving them to a safe environment where the risk to themselves or others are minimized and a prompt evaluation of their current clinical state and the risk for an escalation of their symptoms can be undertaken. The initial evaluation should include consideration of the presence of risk factors for PMA, these may include demographic factors (males, less than 40 years old, low educational level, etc.), psychosocial factors (history of conflict with staff or other patients, a recent stressful life event, involuntary or long-term admission, etc.) or clinical factors (personal or family history of previous agitation episodes, anxiety, fear, substance use, low adherence to treatment, etc.). Several rating scales are available that can be used to evaluate the future risk of agitation, aggression and associated violence in psychiatric inpatient settings. These include the Broset Violence Checklist (BVC [13]), the Historical Clinical Risk Management – 20 (HCR-20 [14]) and the McNiel-Binder Violence Screening Checklist (VSC [15]).

**Fig. 1:**
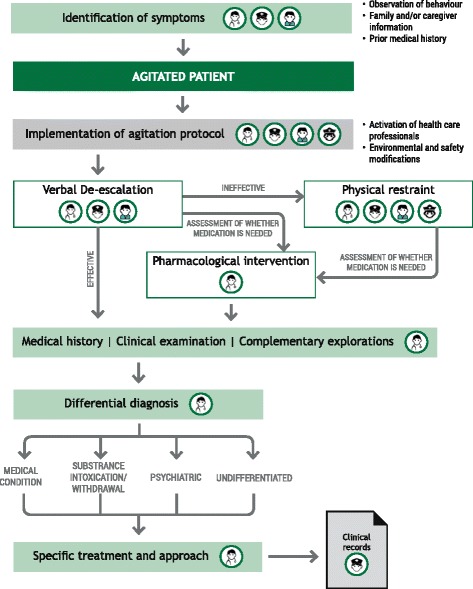
Algorithm for action in agitation. Algorithm for the initial identification and first steps in the management of the patient with psychomotor agitation

Figure 1 provides an algorithm for the initial evaluation and management of the patient with PMA. The initial assessment should be made by two HCPs with experience in the assessment and management of patients with PMA. The aim of the initial assessment is to (1) exclude potential medical causes, (2) achieve rapid stabilization of the patient’s condition, (3) avoid the use of coercive measures, (4) ensure the least restrictive form of management, (5) achieve a therapeutic alliance with the patient and (6) develop an appropriate care plan [2, 8].

The signs and symptoms that may help in the identification of a PMA are shown in Table 1.

**Table 1:** Signs and symptoms of psychomotor agitation [2]

Type	Signs and Symptoms
Changes in behaviour	• Combative attitude • Inappropriate behaviour without clear purpose • Hyperreactivity to stimuli • Inability to remain quiet, seated or calm • Exaggerated gesticulation • Facial tension and angry expression • Defiant and/or prolonged visual contact • Raised tone of voice, silence or refusal to communicate • Altered emotional state with appearance of anxiety, irritability or hostility • Verbal and/or physical aggression against self or others or objects
Cognitive changes	• Fluctuations in the levels of consciousness • Temporo-spatial disorientations • Tendency to frustration • Difficulty in anticipating consequences • Delusional ideas and/or hallucinations
Change in physical parameters	• Fever • Tachycardia • Tachypnoea • Sweating • Tremor • Neurological signs such as difficulty walking

PMA is a clinical phenomenon whose severity is presented as a continuum and so its signs and symptoms. To measure the symptom escalation and severity of agitation, several rating scales have been developed, including the Clinical Global Impression Scale for Aggression (CGI-A; [16]), the Positive and Negative Syndrome Scale – Excited Components (PANSS-EC or PEC; [17]) and the Behavioural Activity Rating Scale (BARS; [18]). The CGI-A relies only on the judgement of the physician and patients can be rapidly evaluated for the severity of their aggressive symptoms using a 5-point Likert scale (1 indicates aggressive behaviour is not present and 5 represents aggressive behaviour is present). The PANSS-EC evaluates 5 items on a scale of 1 to 7 and represents a simple intuitive tool for the evaluation agitated psychiatric patients. Unlike the CGI-A and the PANSS-EC, the BARS measures the severity of agitate behaviour using a single item consisting of 7 levels of severity from a state of sedation to one of agitation. This scale is rapid and easy to administer and does not require medical training.

In addition to the immediate evaluation of the patient’s current state, a medical, toxicological, psychiatric and pharmacological history should be reviewed. Events leading up to the presentation for care should also be considered to identify possible precipitating factors for the current episode of PMA. Physical examination should be undertaken including vital signs, glucose levels, blood oxygen saturation, hepatic and renal function and urine drug test [2]. Electrocardiogram, X-ray of the thorax, lumbar puncture and pregnancy test for females may also be considered [2].

#### Establishing a differential diagnosis

A correct differential diagnosis will help identify the possible underlying cause of the episode of PMA and facilitate the identification of an appropriate management strategy. If the patient’s status does not allow differential diagnosis, a medical condition should be assumed until proven otherwise [2]. PMA due to medical causes typically presents with an acute or subacute onset, frequently in patients of advanced age, without prior psychiatric history and follows a fluctuating course. Such patients tend to exhibit an altered level of consciousness, temporal-spatial disorientation and alteration in physical parameters (sweating, tachycardia, tachypnoea, fever, etc.). Visual hallucinations and delusional ideation as well as cognitive impairment may also be apparent. Due to this, the presence of a confusional state, cognitive impairment, and intoxication/withdrawal syndrome from substances should be considered before considering a psychiatric disorder, especially in cases without past psychiatric history. PMA due to psychiatric causes has generally an acute or subacute onset and presents without alterations in the level of consciousness. If the patient presents with prior psychiatric history, PMA tends to appear in the context of an acute relapse of their mental health disorder. To facilitate a proper management, it has been recommended to differentiate psychotic PMA (associated with schizophrenia, BD) from non-psychotic PMA (associated with anxiety disorders, affective disorders, personality disorders, mental retardation, autism spectrum disorders or adjustment disorders).

If agitation due to medical cause of intoxication/withdrawal syndrome from substances is suspected, supplemental examinations should be requested and the case should be handled in a General Medicine Emergency Department. If the agitation is due to a psychiatric cause, the patient should be treated in a Psychiatric Emergency Department, if it exists at the centre. If the hospital does not have a Psychiatric Emergency Department, the patient should be assisted in the General Medicine Emergency Department.

### Protocol 2: Interventions during an episode of psychomotor agitation

The objectives of an effective management of the PMA, as defined by Zeller and co-workers [8] are to: stabilise the patient quickly; avoid coercive measures; treat in the least restrictive manner; form a therapeutic alliance; and ensure an adequate plan for subsequent care.

Initial interventions should be always attempted in the least restrictive manner: environmental modifications and verbal de-escalation. Then, depending of the severity of the PMA, these techniques might be supplemented by more restrictive options that include pharmacological treatment and/or physical restrain if needed.

#### Environmental modifications

These strategies may be useful to prevent an episode of PMA, to treat an episode during the early stage and to minimize a possible escalation of symptoms [2]. Environmental modifications aim to ensure physical comfort of the patient and reduce external stimuli (irritating factors such as light, noise, cold or hot air currents). Removal of all objects that may be potentially dangerous and maintenance of an optimal safe distance to respect the patient’s personal space should also be undertaken.

#### Verbal de-escalation

This interactive and complex technique is a dynamic process in which the patient is oriented towards a state of calm meanwhile the therapeutic relationship is established [19]. Verbal de-escalation has been shown to reduce PMA and the risk of symptoms escalation as well as to reduce the need for coercive measures [8]. The objectives of verbal de-escalation are: to re-establish patient’s self-control; introduce clear behavioural limits; ensure the safety of the patient, staff, and other users of the healthcare system; achieve a therapeutic alliance with the patient that permits performance of an appropriate diagnostic evaluation; ensure involvement of the patient in his/her own therapeutic decision-making process; and reduce hostility and aggressiveness, preventing possible episodes of violence [8, 19]. While a team approach is needed to manage the patient with PMA, it is recommended that only one person interacts directly with the patient when verbal de-escalation is attempted [19]. This interaction should be calm and concise using simple language, active listening and repetition to establish trust and to identify the patient’s feelings and needs [8]. Table 2 outlines the essential component of a verbal de-escalation technique.

**Table 2:** Essential elements of a verbal de-escalation technique [20]

• Talk with the patient in a gentle, relaxed, assured tone	
• Answer calmly, maintaining a firm attitude	
• Offer food, beverages and blankets	
• Be flexible in the dialogue	
• Reserve your own judgement regarding what the patient should or should not do	
• Do not seek confrontation of ideas or reasons, only simple partnerships that calm and reinforce the patient	
• Use simple language and short sentences, repeating as many times as necessary	
• Be honest and accurate	
• Clearly communicate that the patient is expected to maintain self-control and that the staff can help him/her achieve this	
• Redirect the conversation when disruptive questions are asked	
• Paraphrase what the patient says	
• Reassure the patient that you have understood him/her well	
• Use open-ended questions	
• Establish limits whilst at the same time offering the patient acceptable and realistic opportunities to improve their symptoms	
• When faced with imminent violence: • Warn the patient that violence is not acceptable • Propose a resolution to any problem through dialogue • Offer pharmacological treatment • Inform them that you will rely on physical restraint if necessary	
• Consider a mild/moderate show of force in the form of an increased number of medical staff and even security guards ready to act if necessary	

#### Pharmacological treatment

The primary goal of pharmacological treatment is to rapidly calm the patient without over sedation. Throughout the process, both verbal de-escalation and environmental modification techniques should be maintained.

**Fig. 2:**
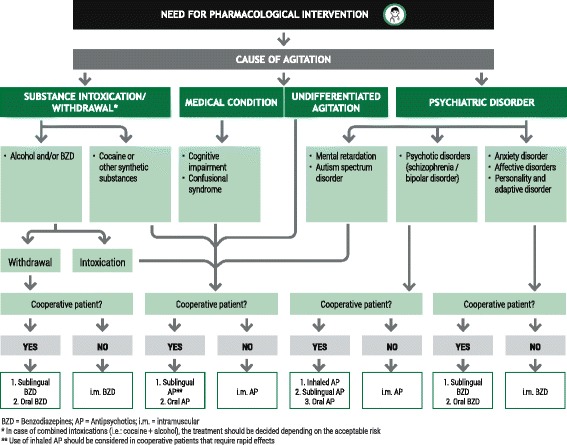
Algorithm for choice of medication. Algorithm for the selection of appropriate pharmacological intervention for the patient with psychomotor agitation

Figure 2 provides an algorithm for the selection of appropriate pharmacological agents guided by the underlying cause and severity of the agitation. All HCPs should be familiar with the available treatments and well trained on when and how to use the different alternatives in order to choose the most convenient one in each case. Ideally, the route of administration should be non-invasive and non-traumatic, to preserve the physician-patient partnership, and the patient should be involved in the decision-making process. Where possible, medication should be given as monotherapy. A rapid onset of action is also a desirable feature of an ideal medication for the treatment of acute PMA.

In case of psychiatric agitation, the preferred pharmacological treatment option if agitation is due to psychotic symptoms is antipsychotic agents although benzodiazepines may also be considered when agitation is due to a non-psychotic agitation [8, 20]. In cases where a rapid effect of the antipsychotic medication is needed, and the patient cooperates, consider a medication with an inhaled route of administration (loxapine; [21, 22]) or an oral/sublingual formulation (olanzapine, risperidone, asenapine, aripiprazole, quetiapine, ziprasidone or haloperidol). Intramuscular (IM) antipsychotic agents (haloperidol, olanzapine [23], ziprasidone [24], aripiprazole [25, 26] and levomepromazine) may be considered for patients who refuse to cooperate with an inhaled or orally administered medication. Despite antipsychotics have been widely used in treating PMA, it has to be noted that some are not indicated for PMA itself but for the possible psychiatric underlined condition (e.g.; oral formulations for olanzapine, risperidone, asenapine, aripiprazole, quetiapine, ziprasone and haloperidol, and the IM formulations of haloperidol and levomepromazine [20]). Table 3 provides an overview of the pharmacological treatment options (approved and not currently approved) for the psychiatric patient presenting with PMA and their suitability with regard to the underlying cause of the PMA.

**Table 3:** Pharmacological treatment options for the patient presenting with psychomotor agitation [21]

Route of administration	Agent	Dose	Cause of agitation
Antipsychotics			
Inhaled	Loxapine	9.1 mg	Psychotic syndrome (schizophrenia, bipolar disorder)
Oral	Olanzapine	5–10 mg	Undifferentiated agitation Medical illness (cognitive deterioration and confusion syndrome) Substance intoxication/abstinence Psychiatric illness (schizophrenia, bipolar disorder, mental retardation and autism spectrum disorder)
	Risperidone	1–3 mg	
	Asenapine	5–10 mg	
	Aripiprazole	15–30 mg	
	Quetiapine	50–100 mg	
	Ziprasidone	20–40 mg	
	Haloperidol	5 mg	
Intramuscular	Haloperidol	5–15 mg	
	Olanazapine	5–10 mg	
	Ziprasidone	10 mg	
	Aripiprazole	9.75 mg	
	Levomepromazine	25 mg	
Benzodiazepines			
Oral	Diazepam	5–10 mg	Abstinence from alcohol and/or BZD Psychiatric illness (anxiety disorder, affective disorder, personality and adjustment disorder)
	Clonazepam	1–2 mg	
	Lorazepam	1 mg	
Intramuscular	Midazolam	5 mg	
	Diazepam	5–10 mg	

*BZD*, benzodiazepine

Caution should be exercised when diagnostic aetiology is not sufficiently clear (undifferentiated agitation) and the patient presents with an altered state of consciousness, in this situation a medical condition for the PMA should be considered until demonstrated to be the contrary [2]. In this regard, both PMA due to medical condition or undifferentiated agitation, should be initially treated with antipsychotic agents. For agitation in patients with Parkinson’s or Parkinson’s-like disorder, the typical antipsychotics (haloperidol, levomepromazine) should be avoided and ziprasidone can be considered as an alternative [2]. For postictal agitation, it may be advisable to use benzodiazepines [2].

When the likely cause of the PMA is related to alcohol and/or benzodiazepine intoxication, caution should be exercised regarding the use of sedatives due to the risk of respiratory depression [2]. Antipsychotics should be considered to avoid the risk of arterial hypertension and respiratory depression. In cases of alcohol and/or benzodiazepine withdrawal, a benzodiazepine should be considered to reduce the risk of seizures and *delirium tremens* [2]. In addition, adding B-vitamin treatment in these patients might also prevent serious complications in alcohol user patients [27]. For cases of cocaine and synthetic drug intoxication, initial sedation with benzodiazepine should be considered instead of antipsychotics in order to decrease the potential risk of seizures [2].

#### Seclusion and physical restraint

Under special circumstances and as a last resource to control de patient, for non-collaborative patients with severe PMA, it might be necessary the use of seclusion and physical restraint to ensure the safety of the patient and the staff and to guarantee pharmacological treatment. These treatment approaches always must be chosen as a last resort treatment. When seclusion is needed, a specially equipped room should be used for seclusion with protective walls and equipped doors to guarantee the reduction of stimuli and the safety of the patient.

Physical restraint is a procedure during which approved mechanical holding devices are used to limit the patient’s physical mobility [3]. Physical restraint is indicated in patients exhibiting risky behaviour towards themselves or those around them, with agitation that cannot be controlled pharmacologically and/or who require temporary restraint to receive the appropriate treatment. Physical restraint should be considered as exceptional and a last recourse when other strategies have failed as this approach could result in negative outcomes for the patient (including physical and mental health negative effects). From the beginning of this process, the patient must be informed about the reason for the restraint, and given a further opportunity to comply with alternative treatment options. It should be explained that the restraint is not a punishment but is intended to ensure their safety.

Physical restraint is a measure limiting the individual’s freedom and, therefore, must be authorized by the patient or the appropriate local authority. In relation to the current Spanish local policies, the physical restraint may be applied as follows:

- Patient admitted voluntarily and with consent for immobilisation: restraint is voluntary or requested by the patient in the event of failure of other measures. Despite this, since they are deprived of liberty, this must be communicated to the local authorities.
- Patient admitted voluntarily but without consent for immobilisation: physical restraint is applied against their will and, although under voluntary admission, it would then be considered involuntary and the local authorities must be informed.
- Patient with involuntary admission: physical restraint is applied against their will in an involuntary admission, where the local authorities have been already awarded of this admission.

The previous clinical status of the patient must be taken into consideration when physical restraint is applied. Situations in which the use of physical restraint is contraindicated include recent eye surgery or neurosurgery, which may result in increased intraocular or intracranial pressure.

**Fig. 3:**
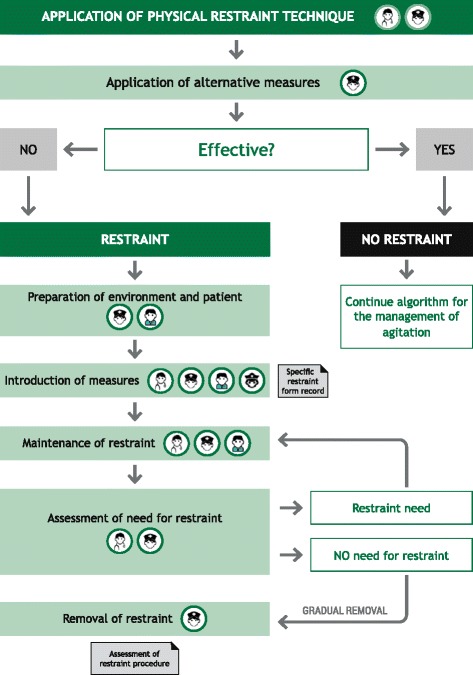
Algorithm for physical restraint. Algorithm for the patient with psychomotor agitation requiring physical restraint

Figure 3 provides an algorithm for the best standards of care and monitoring when physical restraint is needed. Once the decision has been made that physical restraint is required and appropriate (not contra-indicated), staff should remove not only all potentially harmful objects from their person but also any objects that the patient is carrying. The bed must have approved restraint materials and should be at the lowest level possible. No further attempts at verbal de-escalation should be undertaken during the development of the technique. The patient and/or their family should be informed and consent requested although non-consent by the patient does not preclude the application of physical restraint. A single member of staff should communicate with the patient and coordinate the restraint team. If the patient cooperates, they should be escorted to the bed by two personnel holding the patient at the armpit and wrist. Five personnel are required for a non-cooperative patient. They should be guided to the floor and supported by the shoulders and forearms, legs, knees and ankles during transfer to the bed. One person should support their head throughout. Approved restraint devices should be applied to the abdomen and upper and lower limbs.

Because some negative outcomes have been described due to physical restraint, it is essential to closely supervise the patient during the physical restraint including monitoring of general, clinical and safety variables. General monitoring of physical restraint should include direct observation of the patient each 15 min during the first two hours and then hourly until the restraint is removed. If IM medication was administered, monitoring should be performed every 30 min during the first two hours, then at four hours and at six hours. Monitoring should be maintained for as long as the patient is restrained, and for as long as the administration of the IM medication lasts. Other assessments include the patient’s level of consciousness. Changing the patient’s position every hour and the holding devices should be repositioned to reduce the risk of pressure sores and promote circulation and the head of the bed must always be at a 30-degree angle. Throughout the period of restraint, the patient’s basic needs must be met including feeding, hydration, hygiene, elimination, active-passive mobilisation, posture, body alignment, physical and emotional well-being, keeping privacy and frequent communication with the patient.

The restrained patient should be clinically assessed at frequents periods with the goal of removing the restraints as soon as possible. Restraints should be removed gradually with a clear explanation of the behaviour expected of the patient and with at least two HCPs present.

### Protocol 3: After an episode of psychomotor agitation

After resolution of an episode of PMA, it is advisable to debrief what happened with the therapeutic team and with the patient and their family. An adequate review of how the initial identification and care approach, assessment and treatment process unfolded can help the clinical team to better understand what happened, to share comments on relevant variables, to review the action taken and to analyse possible improvements for future episodes [28].

It is advisable to discuss the subjective experience of the patient, what they experienced, and what their ideas and feelings were during their care. The objective of this intervention is to help them to be conscious of how they process reality and their emotional state during the episode of PMA. This process may also help the patient to identify ways to prevent new episodes and establish an agreed treatment plan in which the patient can participate voluntarily [28]. It is also important to enable the patient how to recognise the signs which predict episodes of PMA. In this way, the patient would learn to ask for help earlier if a new episode of agitation appears. It is also advisable to explain to the patient the role of pharmacotherapy in the prevention of symptom escalation [29]. Benefits of this ulterior intervention include the restoration of the therapeutic relationship, decreasing the traumatic nature of some events such as emergency IM injections and decreasing the risk of new episodes of PMA [29]. A strong therapeutic alliance between the patient and the therapeutic team leads to improved long-term control of episodes of PMA.

### Strengths and limitations

The most relevant strengths of this protocol proposal include the development process and the agreement among experts. For the development process, we systematically reviewed the evidence (Jadad’s and PRISMA criteria) and for the agreement among experts, we applied a formal consensus method and collected experts’ opinions (semi structured interviews and external reviews) to reach an agreement for the clinical guidelines related to PMA in adult patients with a primary psychiatric condition. It is relevant to highlight the conducted process of adapting scientific literature to the daily clinical practice in the form of an updated standardized protocol, which is easy to apply in the clinical setting and to replicate. The key limitations of our study are that (i) the consensus methods and convenience samples of current international clinical guidelines [2] may interfere with the Spanish standardized policies, and for extension, with other local regulations; (ii) that there are currently no gold standards for guideline ensuring trustworthy, implementable, and clinically relevant recommendations and (iii) that this protocol would only benefit the agitated patient if it is used and implemented in the clinical practice.

## Conclusions

PMA, a state of motor restlessness and mental tension, requires timely recognition, appropriate initial assessment and management to minimize anxiety for the patient and reduce the risk for escalation to aggression and violence that may be directed towards themselves or others. Protocols and algorithms can assist HCPs to identify patients at risk for PMA, achieve timely diagnosis and implement minimally invasive management strategies to ensure patient and staff safety and resolution of the episode. The protocols and algorithms provided here facilitate this process and provide a structure for the provision of environmental containment and verbal de-escalation with guidance on the appropriate selection and use of pharmacological treatment (inhaled/oral) and seclusion and physical restraint if needed. This protocol represents the application and local adaptation of international consensus guidelines on the standard of care for psychiatric patients suspected of or presenting with PMA within the Spanish healthcare system [2]. Further adaptions may be required for application of this protocol to other healthcare systems.

Implementation of the algorithms described here should ensure that patients at risk for PMA are identified and monitored, episodes of PMA are identified early in their clinical course and that patients can be managed in the least invasive and confrontational way possible, ensuring their own safety and that of others around them. A period of reflection following an episode of PMA can help to re-establish the therapeutic bond between patients and staff and help the patient to recognise their own triggers and identify the early signs of an impending episode. Establishing specialized teams in agitation and providing them with continuing medical education on the identification of agitation, patient management and therapeutic pharmacological alternatives might reduce the burden of PMA for both the patient and the health system.

Regular review and endorsement of PMA protocols and systematic assessment of their level of implementation in the hospital, might have an important effect in reducing the burden of PMA in patients, decrease the need for coercive measures and hospitalizations and especially reinforce patient-physician alliance.

## Abbreviations

BARS: Behavioural Activity Rating Scale
BD: bipolar disorder
BVC: Broset Violence Checklist
CGI-A: Clinical Global Impression Scale for Aggression
CIBERSAM: Spanish Network Centre for Research in Mental Health
HCP: healthcare professional
HCR-20: Historical Clinical Risk Management – 20
IM: intramuscular
PANSS-EC (PE): Positive and Negative Syndrome Scale – Excited Component
PMA: Psychomotor agitation
SEPB: Spanish Society of Biological Psychiatry
VSC: McNiel-Binder Violence Screening Checklist
